# Trends of Multidrug-Resistant Pathogens, Difficult to Treat Bloodstream Infections, and Antimicrobial Consumption at a Tertiary Care Center in Lebanon from 2015–2020: COVID-19 Aftermath

**DOI:** 10.3390/antibiotics10081016

**Published:** 2021-08-21

**Authors:** Amanda Chamieh, Rita Zgheib, Sabah El-Sawalhi, Laure Yammine, Gerard El-Hajj, Omar Zmerli, Claude Afif, Jean-Marc Rolain, Eid Azar

**Affiliations:** 1 Department of Infectious Diseases, Saint George Hospital University Medical Center, Beirut 11002807, Lebanon; amanda.chamieh@gmail.com (A.C.); lyyammine@stgeorgehospital.org (L.Y.); ozmerli@gmail.com (O.Z.); cmafif@stgeorgehospital.org (C.A.); 2 Institut de Recherche pour le Développement (IRD), UMR Microbes Evolution Phylogeny and Infections (MEPHI), Aix-Marseille Université, 13007 Marseille, France; sabah.sawalhi@hotmail.com; 3 Institut Hospitalo-Universitaire Méditerranée Infection, 13005 Marseille, France; zgheibrita@gmail.com; 4 Institut de Recherche pour le Développement (IRD), Service de Santé des Armées, AP-HM, UMR Vecteurs Infections Tropicales et Méditerranéennes (VITROME), Aix Marseille Université, 13007 Marseille, France; 5 Department of Medical Imaging, Saint George Hospital University Medical Center, Beirut 11002807, Lebanon; gerard.hajj@gmail.com

**Keywords:** MDR and COVID-19, CRE, *Acinetobacter baumannii* and COVID-19, VRE and COVID-19, antimicrobial resistance, antimicrobial stewardship in COVID-19

## Abstract

Introduction: We studied the trend of antimicrobial resistance and consumption at Saint George Hospital University Medical Center (SGHUMC), a tertiary care center in Beirut, Lebanon, with a focus on the SARS-CoV-2 pandemic. Materials and Methods: We calculated the isolation density/1000 patient-days (PD) of the most isolated organisms from 1 January 2015–31 December 2020 that included: *E. coli* (Eco), *K. pneumoniae* (Kp), *P. aeruginosa* (Pae), *A. baumannii* (Ab), *S. aureus* (Sau), and *E. faecium* (Efm). We considered March–December 2020 a surrogate of COVID-19. We considered one culture/patient for each antimicrobial susceptibility and excluded *Staphylococcus epidermidis, Staphylococcus* coagulase-negative, and *Corynebacterium* species. We analyzed the trends of the overall isolates, the antimicrobial susceptibilities of blood isolates (BSI), difficult-to-treat (DTR) BSI, carbapenem-resistant *Enterobacteriaceae* (CRE) BSI, and restricted antimicrobial consumption as daily-defined-dose/1000 PD. DTR implies resistance to carbapenems, beta-lactams, fluoroquinolones, and additional antimicrobials where applicable. Results and Discussion: After applying exclusion criteria, we analyzed 1614 blood cultures out of 8314 cultures. We isolated 85 species, most commonly Eco, at 52%. The isolation density of total BSI in 2020 decreased by 16%: 82 patients were spared from bacteremia, with 13 being DTR. The isolation density of CRE BSI/1000 PD decreased by 64% from 2019 to 2020, while VREfm BSI decreased by 34%. There was a significant decrease of 80% in Ab isolates (*p*-value < 0.0001). During COVID-19, restricted antimicrobial consumption decreased to 175 DDD/1000 PD (*p*-value < 0.0001). Total carbapenem consumption persistently decreased by 71.2% from 108DDD/1000 PD in 2015–2019 to 31 DDD/1000 PD in 2020. At SGHUMC, existing epidemics were not worsened by the pandemic. We attribute this to our unique and dynamic collaboration of antimicrobial stewardship, infection prevention and control, and infectious disease consultation.

## 1. Introduction

The SARS-CoV-2 pandemic has greatly marked our past year, and its burden may be reflected in the 3,770,361 deaths worldwide between March 2020 and 11 June 2021 [1]. Unfortunately, it revealed the devastating realities of healthcare systems worldwide, which mostly fell under the pressure of a massive influx of critically ill patients [2,3]. This load of sickness and disease was unprecedented in our day and time, leaving countries unprepared and resorting to instinctual lockdown and quarantine measures in the face of what was an unknown and invisible enemy [4,5,6].

The instantaneous availability of information to the public leads the world to a daily battle of scientific facts, skepticism, and reality versus predictions, myths, social media, and feelings [7,8,9,10]. We embraced cultural changes ranging from lockdowns, social and physical distancing, universal face masks, optimistic cures and vaccines, and lastly, the different strains and mutations of SARS-CoV-2. The duality of an infodemic and a deadly pandemic became our daily bread and butter [11,12,13]. As a multitude of therapeutic regimens arose, so did various reports of collateral damage. A higher number of critically ill patients, insufficient PPE, understaffed hospitals, prolonged hospitalizations, and longer durations of invasive mechanical intubation are all risk factors that may increase nosocomial infections [14,15,16,17,18]. What makes this more concerning is the rising spread of antimicrobial resistance [19,20], especially since nearly all the above-mentioned risk factors may favor harboring multi-drug resistance organisms [21], including last resort options such as carbapenem and colistin.

The concerns about the increase of antimicrobial resistance were reflected in countries with a high burden of severe COVID-19 cases, including the USA, China, France, Iran, and Brazil [22,23,24,25,26,27]. Between 27 January and 17 March 2020, in Wuhan, China, the top causal agents of secondary bacterial infections were *A. baumannii* and *K. pneumoniae* with carbapenem-resistance rates of 91.2% and 75.5%, respectively [22].

In this study, we describe the pathogens isolated at Saint George Hospital University Medical Center (SGHUMC) in Beirut, Lebanon, from 2015 through 2020. We then look at the antimicrobial susceptibilities of the frequently isolated organisms and difficult-to-treat (DTR) bloodstream infections and perform a trend analysis. We highlight the role of COVID-19 on our hospital ecology and on antimicrobial consumption.

## 2. Materials and Methods

### 2.1. Study Design and Data Collection

We conducted this retrospective study at Saint George Hospital University Medical Center (SGHUMC), a 333-bed tertiary care center located in Beirut, Lebanon, from 1 January 2015 to 31 December 2020. Since March 2020, SGHUMC has served as a national COVID-19 referral center for treatment and diagnosis. We have a dedicated COVID-19 inpatient ward, intensive care unit, and emergency fever clinic.

We analyzed the trend and antimicrobial susceptibilities of all isolated bacterial pathogens and bloodstream isolates (BSI) and the trend of consumption of restricted antibiotics. All were calculated as a function of patient days (PD). For this purpose, we accessed our existing SGHUMC antimicrobial stewardship and clinical microbiology electronic database. No medical records were retrieved.

### 2.2. Microbiology

We only considered one culture per patient for each antimicrobial susceptibility interpretation. We included the following organisms that were isolated more than 500 times during the study period: *Escherichia coli* (Eco), *Klebsiella pneumonia* (Kp), *Pseudomonas aeruginosa* (Pae), *Acinetobacter baumannii* (Ab), *Staphylococcus aureus* (Sau), and *Enterococcus faecium* (Efm). As for blood cultures, we excluded *Staphylococcus epidermidis, Staphylococcus* coagulase negative, and *Corynebacterium* species.

### 2.3. Definitions

We define isolate density as the number of isolates/1000 PD (the number of isolates divided by the patient days in a given time multiplied by 1000). We describe carbapenem resistance according to the 2019 CDC definitions and the CLSI. For Sau, we distinguish between methicillin-resistant staph aureus (MRSA) and methicillin-sensitive staph aureus (MSSA) as per the CLSI. As for *E. faecium*, we distinguish between vancomycin-resistant (VREfm) and vancomycin-susceptible. We also calculated the isolate density of difficult to treat (DTR) organisms as previously described [28,29,30]. This implies resistance to carbapenems, beta-lactams, fluoroquinolones, and additional antimicrobials where applicable (i.e., piperacillin-tazobactam, aztreonam in Pae and Ab; vancomycin, linezolid in Sau and Efm).

### 2.4. Antimicrobial Consumption

We calculated the monthly prescribed restricted antimicrobials from 1 January 2010 to 31 December 2021 as daily defined dose (DDD)/1000 patient days (PD), according to the current WHO ATC/DDD guidelines [31]. At SGHUMC, our antimicrobial stewardship program has protocolized a list of restricted antimicrobials that require pre-authorization; this list is regularly updated since 2012 [32]. The restricted antibiotic list includes the following: ertapenem, meropenem, imipenem, colistin, piperacillin-tazobactam, cefepime, ceftazidime, ceftolozane-tazobactam, ceftaroline, vancomycin, linezolid, tigecycline, amikacin, and gentamicin. Aztreonam and intravenous fosfomycin are not available in Lebanon.

### 2.5. Statistical Analysis

The Mann–Kendall trend test and homogeneity test (95% confidence interval (CI); *p*-value < 0.05) were applied on antimicrobial consumptions and isolation densities, both of which were expressed per 1000 patient days to analyze the trend progression over time. Segmented regression analysis for these interrupted time series was performed on RStudio to assess the statistical significance of the different time periods. Best fit linear modeling was applied [33]. All statistical analyses of antimicrobial consumption were done on monthly values per 1000 PD, whereas for isolation densities, we used yearly values. All analyses were performed on Microsoft Excel 2016 with the XLSTAT 2014 add-on [34]. We used the non-parametric Kruskal–Wallis test with a two-sided *p*-value < 0.05 as the significance level.

## 3. Results

### 3.1. General Results and Species Repartition

We retrieved a total of 14,005 individual bacterial cultures during the study period, 3726 of which were blood cultures. After applying exclusion criteria, we analyzed a total of 8314 bacterial cultures, of which 1614 were blood cultures. Eighty-five different species were isolated (Supplementary Table S1), of which we studied the following organisms that were isolated more than 500 times in our laboratories: *Escherichia coli* (Eco), *Klebsiella pneumonia* (Kp), *Pseudomonas aeruginosa* (Pae), *Acinetobacter baumannii* (Ab), *Staphylococcus aureus* (Sau), and *Enterococcus faecium* (Efm). A total of 453,307 patient days were recorded throughout the study period.

The most isolated organism was Eco, constituting an average of 51% of all isolates from 2015–2019, which increased to 53% in 2020 but with no statistical significance. As for Kp, the second most isolated organism, the average percentage of the isolates increased from an average of 15% from 2015–2019 to 18% in 2020, also with no statistical significance. No significant changes were found with *P. aeruginosa* (13%), *S. aureus* (7–8%), and *E. faecium* (6–7%) isolates. In summary, all isolates maintained almost the same percentage distribution of total isolates over the years, except for Ab, which decreased by 75% from 2015 to 2020, from a peak of 12% in 2015.

### 3.2. Trend Analysis of Isolation Densities of All Organisms

There was no significant trend detected in the isolation densities of Eco from 2015 to 2020, with an average of 9.34/1000 PD (*p*-value = 1). The peak isolation density of Eco was 10.36/1000 PD in 2019, which decreased by 10% to 9.31/1000 PD in 2020. As for Kp, an increasing trend was detected in 2017 (*p*-value < 0.0001) from 2.51/1000 PD in 2015 to a peak of 3.24/1000 PD in 2019. However, in 2020, the isolation density decreased by 3% to 3.15/1000 PD. The isolation density of Pae remained unchanged at an average of 2.31/1000 PD from 2015–2020. To note, the lowest isolation density of Pae of 2.20/1000 PD was in 2020, a decrease of 8%. As for Sau, no significant trend was detected with an average isolation density of 1.33/1000 PD from 2015–2020. Similarly, the isolation density decreased by 22% from a peak of 1.61/1000 PD in 2019 to 1.25/1000 PD in 2020. The isolation density of *Enterococcus faecium* decreased from 1.56/1000 PD in 2015 to an all-time low of 0.99/1000 PD in 2020 (*p*-value < 0.0001). Specifically, there was a decrease of 12.4% in isolation density of Efm from 2019 to 2020. As for Ab, there was a significant decreasing trend from a peak of 2.4/1000 PD in 2015 to 0.55/1000 PD in 2020, a total decrease of 77% from 2015 to 2019 (*p*-value < 0.0001) and a decrease of 15% from 2019 to 2020.

### 3.3. Trend Analysis of Isolation Densities of BSI

There was no significant trend detected in the isolation density of all BSI from 2015 until 2020, with an average of 2.34/1000 PD. However, isolation density decreased by 16%, dropping from 2.73/1000 PD in 2019 to 2.3/1000 PD in 2020.

**Figure 1:**
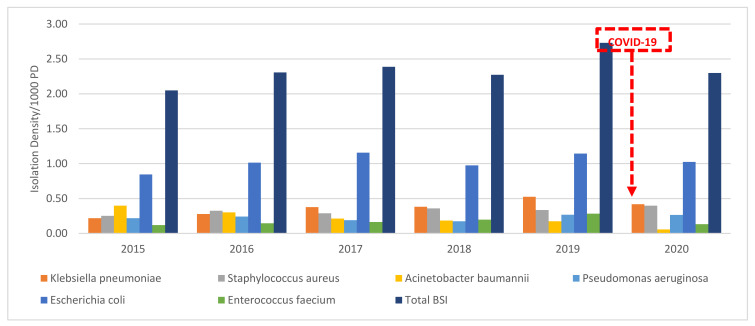
The isolation density per 1000 PD of bloodstream isolates from 2015–2020 at SGHUMC.

There was no significant trend detected in the BSI isolation densities of Eco from 2015 to 2020, with an average of 1.03/1000 PD. The BSI isolation density of Eco was 1.14/1000 PD in 2019, which decreased by 9.6% to 1.03/1000 PD in 2020. As for Kp, beginning in 2017, there was an increasing trend detected (*p*-value < 0.0001) to a peak of 0.52/1000 PD in 2019. However, in 2020, the isolation density decreased by 19% to 0.42/1000 PD. The BSI isolation density of Pae remained unchanged at an average 0.23/1000 PD from 2015–2020. As for Sau BSI, a significant increasing trend was detected with an average isolation density of 0.33/1000 PD from 2015–2020. However, the isolation density increased by 17% to reach a peak of 0.4/1000 PD in 2020. There was no statistically significant trend detected for the BSI isolation density of Efm. Interestingly, there was a decrease of 54% from the peak of 0.28/1000 PD in 2019 to a near all-time low of 0.13/1000 PD in 2020 (*p*-value < 0.0001), the same as in 2015. As for Ab, there was a significant decreasing trend from a peak of 0.4/1000 PD in 2015 to 0.06/1000 PD in 2020, the least isolated BSI in 2020. This represented a decrease of 85% from 2015 to 2020 (*p*-value < 0.0001) and a decrease of 65% from 2019 to 2020 (Figure 1).

### 3.4. Difficult-to-Treat Blood Stream Infections

The total number of difficult-to-treat (DTR) BSI did not significantly change from 2015 until 2019 (a total of 124 isolates and an average of 24.8 isolates per year). However, from 2019 to 2020, there was a significant decrease by 52% of total DTR BSI (from *n* = 27 isolates in 2019 to *n* = 14 in 2020: *p*-value < 0.0001). This corresponds to an average DTR BSI isolation density of 0.31/1000 PD from 2015–2019 to 0.27/1000 PD in 2020. The DTR BSI isolation density of 0.36/1000 PD in 2019 decreased by 25% to 0.27/1000 PD in 2020.

**Figure 2:**
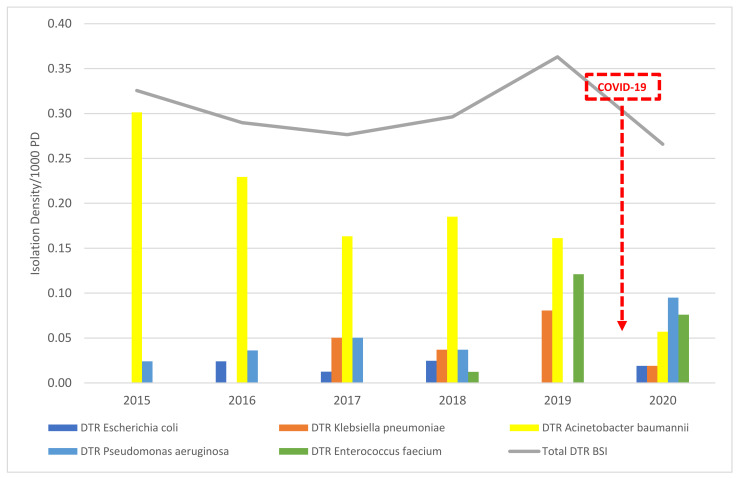
The isolation density per 1000 PD of difficult-to-treat (DTR) bloodstream isolates from 2015–2020 at SGHUMC.

No statistically significant overall trend was detected for DTR BSI Eco, with an average of 0.01/1000 PD from 2015–2020. Kp DTR BSI reached a peak in 2019 of 0.08/1000 PD (*n* = 6), which decreased by 75% to 0.02/1000 PD (*n* =1) in 2020. As for Pae DTR BSI, there was a statistically insignificant increase from an average of 0.03/1000 PD (*n* = 3) from 2015–2019 to 0.09/1000 PD (*n* = 5) in 2020. There was no Sau DTR BSI throughout the study period. Interestingly, Efm DTR BSI increased from 0 in 2015–2017 to a peak of 0.12/1000 PD (*n* = 9) in 2019. However, in 2020, it decreased by 33%, reaching 0.08/1000 PD (*n* = 4) in 2020. There was a significant decrease by 80% in Ab isolates from a peak of 0.3/1000 PD (*n* = 25) DTR BSI isolates in 2015 to only 0.06/1000 PD (*n* = 3) in 2020 (*p*-value = 0.02). In 2019, DTR BSI Ab was 0.16/1000 PD (*n* = 12), and this decreased by 62% to 0.06/1000 PD (*n* = 3) in 2020. Therefore, from 2019 to 2020, 9 patients were spared from Ab DTR BSI (Figure 2).

### 3.5. Carbapenem-Resistant E. coli and K. pneumoniae

**Figure 3:**
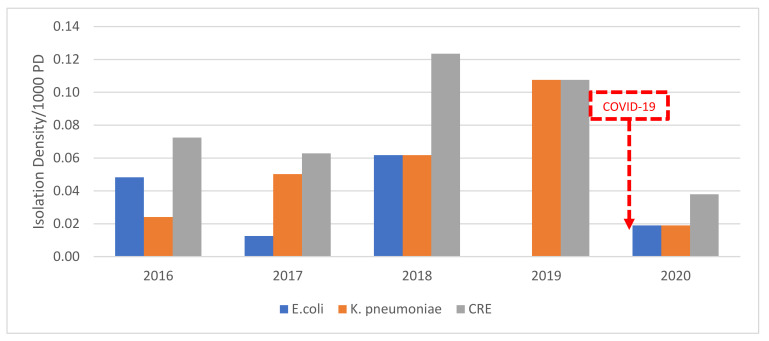
The isolation density per 1000 PD of carbapenem-resistant *E. coli* and *K. pneumoniae* bloodstream isolates from 2015–2020 at SGHUMC.

There was no statistically significant change in the percentage of carbapenem-resistant Eco (CREco) BSI throughout the years. In 2015 and 2019, there were no carbapenem-resistant Eco BSI. In 2015–2018, we have an average of 0.04/1000 PD (*n* = 3; 4% of Eco BSI) CREco BSI. In 2020, we have an average of 0.02/1000 PD (*n* = 1; 2% of Eco BSI) CREco BSI. Despite an insignificant increase, it is still at a lower value than previous years. As for carbapenem-resistant Kp (CRKp) BSI, there is a steady increase starting in 2016 from 0.02/1000 PD (*n* = 2; 9% of Kp BSI) to a peak of 0.11/1000 PD (*n* = 8; 21% of Kp BSI) in 2019. This decreased by 82% to reach an average of 0.02/1000 PD (*n* = 1; 5% of Kp BSI) in 2020. Overall, isolation density of carbapenem-resistant *Enterobacteriaceae* (CRE) BSI in 2020 reached its lowest value since 2016 at 0.04/1000 PD (*n* = 2). This is a decrease of 64% in isolation density of CRE BSI/1000 PD from 2019 to 2020 (Figure 3).

### 3.6. MSSA vs. MRSA BSI

Throughout the study, no statistically significant trends were detected for both MSSA and MRSA BSI. However, there was a decrease in MSSA BSI from 64% in 2015–2019 to 38% in 2020, while mirrored by an increase of MRSA BSI from 36% to 62% in 2020.

In 2015–2019, we had an average of 0.20/1000 PD MSSA BSI that decreased to 0.15/1000 PD in 2020. As for MRSA BSI, we had an average isolation density of 0.11/1000 PD in 2015–2019 that increased to 0.25/1000 PD. However, the increase in MRSA BSI remained statistically insignificant, with only a minor overall increase in Sau BSI.

### 3.7. E. faecium and Vancomycin-Esistant E. faecium BSI

**Figure 4:**
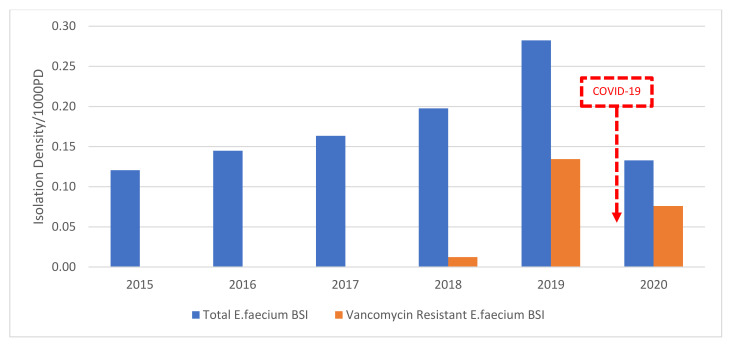
The isolation density per 1000 PD of total *E. faecium* and Vancomycin-resistant *E. faecium* bloodstream isolates from 2015–2020 at SGHUMC.

From 2015–2017, there was an average of 0.14/1000 PD (*n* = 12) Efm BSI, with no vancomycin resistance. In 2018, there was an increase to 0.20/1000 PD of Efm BSI and 0.01/1000 PD (*n* = 1) VREfm BSI. In 2019, we have a peak of 0.28/1000 PD Efm BSI and 0.13/1000 PD (*n* = 10) VREfm BSI. In 2020, we have a decrease of 54% of Efm BSI reaching 0.13/1000 PD (*n* = 7) and a decrease of 39% of VREfm BSI reaching 0.08/1000 PD (*n* = 4). In other words, we have a decrease of 6 cases of VREFm BSI in 2020 (Figure 4).

### 3.8. Antimicrobial Consumption

**Figure 5:**
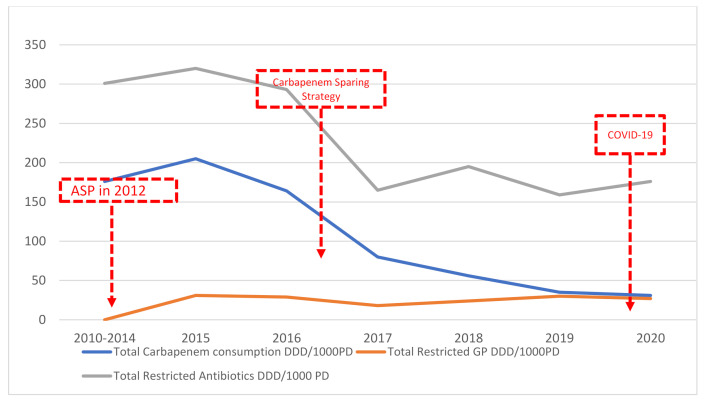
The restricted antimicrobial consumption calculated as daily define dose (DDD) per 1000 PD from 2015–2020 at SGHUMC. In 2012, the antimicrobial stewardship program’s (ASP) electronic pre-approval authorization was implemented at SGHUMC.

The average monthly total restricted antimicrobial consumption for the year 2015 was at a peak of 320DDD/1000 PD, which significantly decreased by 45% to 176 DDD/1000 PD (*p*-value < 0.0001). The average consumption before COVID-19, from 2015–2019, was 226DDD/1000 PD, decreasing to 176DDD/1000 PD in 2020. Total carbapenem consumption persistently decreased by 71.2%, from 108DDD/1000 PD in 2015–2019 to 31 DDD/1000 PD in 2020. This was related to a hospital-wide carbapenem sparing strategy and colistin monotherapy for *A. baumannii* that was implemented in 2016 at SGHUMC [35]. Total restricted antimicrobials targeting Gram-positive organisms were relatively constant over the years, with a minor increase that is statistically insignificant. However, segmented regression analysis did not demonstrate a statistical significance of before and after COVID-19, probably due to the short time span (Figure 5).

## 4. Discussion

In this article, we studied the most isolated pathogens at Saint George Hospital University Medical Center (SGHUMC) in Beirut, Lebanon, from 2015 through 2020. The study included a total of 8314 nonduplicate, clinical bacterial isolates. Out of 85 different species, we analyzed *Escherichia coli* (Eco), *Klebsiella pneumonia* (Kp), *Pseudomonas aeruginosa* (Pae), *Acinetobacter baumannii* (Ab), *Staphylococcus aureus* (Sau), and *Enterococcus faecium* (Efm). We also determined the various trends of isolation density of a total of 1614 unique bloodstream isolates and their antimicrobial susceptibilities and calculated the consumption of restricted antimicrobials. The species repartition over the years remained almost the same, with the exception of Ab, which significantly decreased by 75% from 2015 to 2020, from a peak of 12% in 2015.

In general, there was a decrease in the isolation density of total BSI from 2019 to 2020 by 16%. There was also a significant decrease of 52% of total DTR BSI, from 27 to 14 episodes of DTR BSI from 2019 to 2020. This means that 82 patients were spared from bacteremia, and 13 patients were spared from a DTR bacteremia. This may be one of the indirect outcomes of the COVID-19 pandemic, even if the patient population varied.

Despite the lack of statistical significance in the overall trend analysis of isolates, it is undeniable that there is a difference between 2019 (pre-COVID-19) and 2020 (during COVID-19). We observed a decrease in the total isolation density of Eco (by 10%) and Kp (by 3%). Moreover, we had a 9.6% decrease in Eco BSI isolation density with a 19% decrease in Kp BSI. We also noted a tremendous decrease of 64% in isolation density of carbapenem-resistant Eco and Kp BSI from 2019 to 2020. During 2017, we witnessed an outbreak of carbapenem-resistant *Enterobacteriaceae* [36], regardless of significant curbing of carbapenem. Specifically, carbapenem-resistant Kp BSI decreased by 82% alone. As for difficult-to-treat BSI, Eco DTR BSI was unchanged while Kp DTR BSI decreased by 75%, which translates into a drop from 6 to 1 patient with a DTR Kp BSI. Did the COVID-19 pandemic have a role in curbing and decreasing the burden of our CRE outbreak?

On the other hand, the peak of 0.12/1000 PD (*n* = 9) DTR Efm BSI in 2019 clearly indicated that we were experiencing an outbreak at the time. However, in 2020, it decreased by 33%, implying a drop from 9 to 4 patients with DTR Efm BSI, contrary to reports that have shown VRE outbreaks during COVID-19 [37].

As for Sau, no DTR BSI was recorded throughout the study period, and we had an overall decrease by 22% in the isolation density of Sau from 2019 to 2020. Thus, despite the non-statistically significant increase of the isolation density of MRSA BSI, we still had limited, treatable Sau infections.

There was a significant decrease of 80% in Ab isolates, and, from 2019 to 2020, 9 patients were spared from an Ab DTR BSI while only 3 were affected. In 2016, we introduced a colistin-monotherapy protocol for all Ab infections and implemented a carbapenem sparing strategy throughout the hospital. This eliminated our Ab ST2 clonal outbreak and favorized a wild-type, sensitive Ab strain [35]. This contradicts with recent works completed during COVID-19 to show that, for example, in Iran, *A. baumannii* was the most common organism detected in COVID-19 patients with co-infections [24]. We can assume that in our work, this effect was maintained throughout the COVID-19 pandemic, even resulting in a lower isolation density of Ab than in previous years [38].

To further assess the impact of COVID-19 on our hospital ecology and the apparent decrease in antimicrobial resistance, we performed a trend analysis on our restricted antimicrobial consumption. We noted a significant decrease in consumption of restricted antimicrobials, indicating that there was a true decrease in the need to treat severe infections (Figure 5).

It is expected that functioning under conditions of increased critical care capacities would lead to an increase in nosocomial infections and antimicrobial resistance. As we have all become familiar, during the COVID-19 pandemic, all hospitals operated on a minimal number of staff, no visitors were allowed, and very strict hand hygiene, infection prevention, and control measures were applied [39,40]. This decreased traffic and unnecessary population of the hospital, which may have positively contributed to improving the overall hospital ecology.

Surprisingly, at Saint George Hospital University Medical Center, our already existing epidemics, such as carbapenem-resistant *Enterobacteriaceae* and vancomycin-resistant *Enterococci,* were not worsened by the pandemic. In addition, other epidemics such as carbapenem-resistant *A. baumannii* continued to improve. We attribute this to our unique and dynamic collaboration of antimicrobial stewardship, infection prevention and control, and infectious disease consultation. Invariably, we highlight the role of antimicrobial stewardship and the practice of good, sound medicine during the busiest and most health demanding times our healthcare systems are experiencing.

The presence of multiple confounding variables makes it difficult to quantify and establish a direct causality of the possible positive effect on antimicrobial resistance, which was the main limitation of our study. It is also important to continue to further monitor and survey the isolation density of antimicrobial resistance and antimicrobial consumption to determine the sustainability of probably the only positive impact of COVID-19, a pandemic that may have ended other local epidemics.

## Data Availability

The data presented in this study are available in supplementary material.

## Supplementary Materials

The following are available online at https://www.mdpi.com/article/10.3390/antibiotics10081016/s1, Table S1: All isolated species from 2015–2020 by increasing frequency, Table S1b: Total isolates/1000PD, all sample types.
