# Supplementary material for: Trends of Multidrug-Resistant Pathogens, Difficult to Treat Bloodstream Infections, and Antimicrobial Consumption at a Tertiary Care Center in Lebanon from 2015–2020: COVID-19 Aftermath
Source: Antibiotics (Basel). 2021 Aug 21;10(8):1016. doi: 10.3390/antibiotics10081016 (PMC8388970; doi:10.3390/antibiotics10081016)
Supplement: Supplementary file 1 [file antibiotics-10-01016-s001.zip › antibiotics-1311414-supplementary.pdf]

Supplementary Table S1. All isolated species from 2015-2020 by increasing frequency

Organism	Number of Isolates	2015	2016	2017	2018	2019	2020
Escherichia coli	4239	817	767	722	673	770	490
Staphylococcus epidermidis	1613	267	312	240	273	292	229
Klebsiella pneumoniae ss. pneumoniae	1265	208	208	206	236	241	166
Pseudomonas aeruginosa	1083	237	189	177	185	179	116
Staphylococcus, coagulase negative	679	130	99	88	113	141	108
Staphylococcus aureus ss. aureus	625	127	94	93	125	120	66
Enterococcus faecium	580	129	106	102	107	84	52
Acinetobacter baumannii	522	199	115	73	58	48	29
Proteus mirabilis	472	91	73	74	104	73	57
Enterococcus faecalis	419	47	51	66	51	137	67
Enterobacter sp.	408	128	80	100	68	25	7
Stenotrophomonas maltophilia	282	79	54	37	48	34	30
Morganella morganii ss. morganii	260	63	51	57	47	25	17
Pseudomonas sp.	133	44	27	22	13	17	10
Klebsiella oxytoca	115	23	25	19	20	21	7
Enterobacter cloacae	114	3	2		19	47	43
Citrobacter freundii	105	23	21	18	21	13	9
Salmonella sp.	100	22	15	18	23	14	8
Streptococcus viridans, alpha-hem.	100	13	22	25	24	12	4
Corynebacterium sp.	84	23	12	10	24	14	1
Serratia sp.	84	22	20	15	15	8	4

Streptococcus pneumoniae	70	25	16	7	12	6	4
Citrobacter koseri (diversus)	68	9	11	9	15	13	11
Haemophilus influenzae	68	10	12	20	4	17	5
Proteus vulgaris	52	19	11	11	5	3	3
Streptococcus, beta-haem. Group B	47	15	11	4	12	3	2
Serratia marcescens	43	3	4	2	3	12	19
Klebsiella aerogenes	42		1		14	14	13
Acinetobacter lwoffii	37	13	10	8	2	1	3
Streptococcus mitis	35		4	2	11	12	6
Streptococcus, beta-haem. Group A	35	8	7	6	11	3	
Aerococcus viridans	20	10	8	1	1		
Salmonella Typhi	17	5	6	1	2	2	1
Burkholderia cepacia	15	8	1		5	1	
Raoultella terrigena	15	4		5	6		
Non-fermenting Gram negative rods	14	11	1		1	1	
Streptococcus agalactiae	12				2	2	8
Streptococcus sp.	11				3	4	4
Campylobacter sp.	10	1	3	1	2	3	
Acinetobacter junii	8	1	2	2	2		1
Streptococcus, beta-haem. Group G	8	2	2	1	1	2	
Moraxella (Branh.) catarrhalis	7	5		1	1		
Klebsiella sp.	5		1	1	1		2
Streptococcus, Group D (non-enterococcal)	5	1	1		3		
Aeromonas hydrophila	4	1	1	1		1	
Brucella sp.	4		1	1	2		
Streptococcus bovis	4					3	1

Streptococcus salivarius	4				2	2	
Acinetobacter haemolyticus	3					3	
Enterococcus sp.	3					3	
Escherichia sp.	3	1	1			1	
Pseudomonas putida	3		1	2			
Salmonella Paratyphi B	3	1	2				
Streptococcus gallolyticus ss. gallolyticus	3			1			2
Streptococcus oralis	3			1	2		
Achromobacter sp.	2				2		
Enterococcus gallinarum	2					2	
Gram positive rods	2	1		1			
Haemophilus parainfluenzae	2		1				1
Leuconostoc sp.	2	1		1			
Providencia stuartii	2				1	1	
Stenotrophomonas sp.	2		1	1			
Streptococcus anginosus	2			1			1
Streptococcus bovis II	2	1			1		
Streptococcus constellatus	2						2
Streptococcus pyogenes	2				2		
Streptococcus, beta-haem. Group C	2			1	1		
Achromobacter xylosoxidans ss. xylosoxidans	1				1		
Aeromonas sp.	1					1	
Aeromonas veronii biovar sobria	1					1	
Citrobacter sp.	1				1		
Corynebacterium sp. (diphtheroids)	1				1		

Hafnia alvei	1				1		
Pantoea agglomerans	1				1		
Pasteurella sp.	1					1	
Prevotella oralis	1					1	
Proteus sp.	1					1	
Providencia rettgeri	1					1	
Sphingomonas paucimobilis	1				1		
Staphylococcus lugdunensis	1					1	
Staphylococcus sp.	1			1			
Streptococcus dysgalactiae ss. dysgalactiae	1						1
Streptococcus gordonii	1						1
Streptococcus milleri	1		1				
Streptococcus, beta-haemolytic	1	1					

Supplementary Table S1b: Total Isolates/1000PD , all sample types

Organism	2015	2016	2017	2018	2019	2020
Escherichia coli	9,9	9,3	9,1	8,3	10,4	9,3
Klebsiella pneumoniae	2,5	2,5	2,6	2,9	3,2	3,2
Pseudomonas aeruginosa	2,9	2,3	2,2	2,3	2,4	2,2
Staphylococcus aureus	1,5	1,1	1,2	1,5	1,6	1,3
Enterococcus faecium	1,6	1,3	1,3	1,3	1,1	1,0
Acinetobacter baumannii	2,4	1,4	0,9	0,7	0,6	0,6
